# Acute Effects of Plantar Proprioceptive Training on Dynamic Balance and Ankle Range of Motion: A Pilot Randomized Controlled Trial

**DOI:** 10.3390/sports14050180

**Published:** 2026-05-01

**Authors:** Alberto Canzone, Jessica Brusa, Valerio Giustino, Francesco Martines, Pietro Salvago, Simona Pajaujiene, Antonino Patti, Daniele Zangla, Giuseppe Messina, Elvira Padua, Antonino Bianco

**Affiliations:** 1 Sport and Exercise Sciences Research Unit, Department of Psychology, Educational Science and Human Movement, University of Palermo, 90144 Palermo, Italy; alberto.canzone@univr.it (A.C.); antonino.patti01@unipa.it (A.P.); daniele.zangla@unipa.it (D.Z.); antonino.bianco@unipa.it (A.B.); 2 Department of Neuroscience, Biomedicine and Movement Sciences, University of Verona, 37124 Verona, Italy; 3 Department of Human Sciences and Promotion of the Quality of Life, San Raffaele University, 00166 Rome, Italy; brusajessica@gmail.com (J.B.); giuseppe.messina@uniroma5.it (G.M.); elvira.padua@uniroma5.it (E.P.); 4 Department of Biomedicine, Neuroscience and Advanced Diagnostics (BiND), Section of Audiology, University of Palermo, 90127 Palermo, Italy; francesco.martines@unipa.it (F.M.); pietro.salvago01@unipa.it (P.S.); 5 Department of Coaching Science, Lithuanian Sports University, LT-44221 Kaunas, Lithuania; simona.pajaujiene@lsu.lt

**Keywords:** posture, balance, postural control, proprioception, tonic postural system, sensorimotor integration

## Abstract

Background: An important role in postural control is played by the plantar proprioceptive inputs, as they contribute to the sensorimotor integration of the Tonic Postural System (TPS). Although plantar stimulation is an excellent strategy for improving balance, evidence remains limited. Therefore, the aim of this pilot study was to examine the acute effects of plantar proprioceptive training on dynamic balance performance and ankle range of motion (ROM). Methods: In this randomized controlled trial, 26 physically active young adults were divided into an experimental group (EG; n = 13) and a control group (CG; n = 13). The EG performed plantar proprioceptive training including walking on a reflexology mat and balance exercises on a proprioceptive pad. The CG remained lying supine on a couch for the same amount of time as the experimental intervention. The Y-Balance Test (YBT) was used to assess dynamic balance, while the ankle ROM (i.e., dorsiflexion and plantarflexion) was measured using an inertial sensor. All measurements were taken before (T0) and immediately after (T1) the experimental or control condition. Results: Improvements in the YBT were found in the EG from T0 to T1 for both right (*p* = 0.002; SE = 1.24) and left (*p* = 0.015) foot, but no changes from T0 to T1 were observed in the CG for both right and left foot (*p* > 0.05). No changes were observed for ankle ROM in both groups (*p* > 0.05). Conclusions: These preliminary results suggest that plantar proprioceptive training can provide acute improvements in dynamic balance with no significant changes in ankle ROM. The findings support a potential role of plantar stimulation in postural control mechanisms.

## 1. Introduction

Posture is an automatic mechanism that represents the body’s response to the force of gravity [1]. It is sustained by the contraction of skeletal muscles, which are coordinated through multiple stimuli of different origins and by continuous neuromuscular adjustment [2]. A complex system, commonly referred to as the Tonic Postural System (TPS), manages postural control. The TPS consists of an afferent pathway, which transfers information to the Central Nervous System (CNS), that modulates the posture through an effector pathway [2,3]. In detail, the somatosensory system transmits inputs from internal and external sensory organs, including proprioceptive and exteroceptive receptors, to the CNS [4,5,6]. Somatosensory inputs originate from sensory receptors located at different anatomical levels: (a) muscular—neuromuscular spindles and Golgi tendon organs provide the proprioceptive information; (b) visual—oculomotor organs transmit information about the movement of the visual field and the orientation of the head according to the perceived vision, which is transmitted and detected by the retina; (c) vestibular—otoliths organs transmit information relating to the acceleration and deviation of the head and play a role in controlling postural fluctuations; (d) skin detects the flexion of the foot associated with the support surface, using skin receptors, localized primarily at the level of the foot [1,2,7,8].

Building upon the previously described role of the somatosensory system within the TPS, a fundamental component of postural regulation is represented by proprioceptive information. Proprioception is defined as the ability to integrate sensory signals originating from numerous mechanoreceptors to determine body position and movement in space, and it plays a key role in postural control [9,10,11,12,13].

Concerning skin, this component includes a wide variety of receptors capable of detecting mechanical, nociceptive, or thermal stimuli. These receptors include both bare and encapsulated nerve endings, including Pacinian corpuscles (for detecting vibrations or brief touches), Merkel’s discs (for detecting prolonged deformations or prolonged touches to the skin), Meissner’s corpuscles (for detecting moving touch), Ruffini’s endings (for detecting constant pressure applied to the skin), hair follicle receptors, and Krause’s end bulbs [14]. In this context, it is important to distinguish between the epidermis and the dermis, which have different embryological origins [15]. The epidermis has a key role in pain perception through cutaneous sensory and autonomic nerve fibers [15,16,17], while the dermis is a layer of connective tissue located between the epidermis and the subcutaneous tissue with the role of supporting and protecting the skin and underlying layers, contributing to thermoregulation and increasing sensitivity [16,17,18].

Postural control depends on managing constant body sway to maintain or achieve postural stability. In this way, information from the plantar dermis allows for the management of sway for postural control. As reported below, numerous studies have investigated the effects of plantar dermis stimulation on postural control. In particular, it has been suggested that postural control can be improved by increasing information from the plantar dermis, enhancing the perception of plantar sensory stimuli and facilitating postural responses [19,20,21,22,23,24]. Indeed, the foot plays a key role in transmitting sensory information from the ground to the CNS because it is the direct interface between the body and the ground [20]. In detail, plantar dermis feedback is given by receptors, mentioned above, that exhibit high sensitivity to forces applied to the plantar surface [25,26,27]. Each of these receptors is characterized by adaptive properties and responds to moderate mechanical stimuli, encoding reversible skin deformations which include vibration and pressure [20,28,29,30]. Hence, plantar dermis afferents transmit variations that occur under the foot related to space and time [20,31], providing precise and continuously updated sensory information to the CNS that supports the fine regulation for postural adjustment. In fact, the CNS receives and integrates this information by modulating muscle activity. As a matter of fact, it is responsible for the regulation of muscle tone of the antigravity muscles that allows the maintenance of body posture [2].

In a recent meta-analysis, it has been shown that proprioceptive training induces benefits in terms of balance in subjects with chronic ankle instability (CAI). However, the results did not exceed the minimum detectable change, and the limited number of studies as well as the lack of long-term follow-up studies prevented definitive conclusions from being drawn [32]. In a network meta-analysis, which aimed to compare the effects of different exercise interventions on proprioception in subjects with CAI, the authors found that strengthening the muscles of the foot and ankle can have a positive effect on improving proprioception, and that the more complex the balance exercises become, the less effective the results are [33]. The aforementioned studies have focused primarily on general proprioceptive training and strengthening exercises, while the specific contribution of plantar proprioceptive stimulation remains poorly understood. Furthermore, limited attention has been given to its effects on dynamic balance performance and its potential interaction with ankle joint mobility, which represents a relevant but still unexplored aspect.

Therefore, the aim of this pilot study was to examine the acute effects of plantar proprioceptive training on dynamic balance performance and ankle range of motion (ROM) in physically active young adults. In particular, ankle dorsiflexion and plantarflexion were measured to explore potential changes in joint mobility associated with the plantar proprioceptive stimulation. Based on the role of plantar proprioceptive input in postural control regulation, it is hypothesized that plantar proprioceptive training may lead to improvement in dynamic balance performance rather than inducing immediate changes in ankle mobility.

## 2. Materials and Methods

### 2.1. Study Design

This pilot study was designed as an open-label randomized controlled trial. Participants were randomly allocated to either an intervention group or a control group. The allocation ratio was 1:1. The experimenter was aware of group allocation, while participants were informed of their group assignment on the day of the intervention. No changes to the study methods, eligibility criteria, or outcome measures were made after trial commencement.

### 2.2. Participants

The following criteria were taken into consideration for the study: age between 20 and 30 years; absence of postural disorders (e.g., scoliosis); people without musculoskeletal injuries in the three months preceding the start of the study (e.g., muscle/tendon/ligament tears); absence of neurological disorders (e.g., epilepsy); and no presence of vestibular disorders (e.g., vertigo). In detail, the absence of postural, musculoskeletal, and neurological disorders was self-reported, while the absence of vestibular disorders was clinically assessed. In particular, a complete neuro-otological visit was administered by two medical doctors specialized in audiology (F.M. and P.S.).

Data were collected in the Laboratory of Posturology and Biomechanics of the University of Palermo. The period during which the data collection was carried out was between January and February 2026, with testing sessions planned between 08:00 and 14:00 and between 15:00 and 17:00. Participants were enrolled through direct contact, and after voluntarily agreeing to participate in the study, they all provided written informed consent before participation, and received detailed information about the study procedures.

The study was approved by the Bioethics Committee of the University of Palermo on 18 December 2025 (n. 355/2025—prot. 206649-2025 and 227550-2025).

### 2.3. Intervention

#### 2.3.1. Experimental Group (EG)

The experimental protocol combined walking and balance exercises. The following exercises were performed:

Exercise 1—Walking on a plantar reflexology mat: participants walked forward on a plantar reflexology mat for three sets of 1 min each with 30 s of rest between sets. The reflexology mat measured 2.50 m. After the conclusion of the first exercise, a rest period of 30 s was allowed for participants before the next exercise.

Exercise 2—Bipodal stance on two proprioceptive cushions: a bipodal position on two inflatable proprioceptive cushions filled with air was maintained by participants. The cushions had two different surfaces, one with thicker pegs and one with thinner pegs, and both were used. As the first exercise, participants executed three sets of 30 s on the surface with thicker pegs, with 30 s of rest between sets, followed by three sets of 30 s on the surface with thinner pegs, with 30 s of rest between sets as the second exercise. During this exercise, participants were told to maintain balance while visually fixating on a red dot (4 cm diameter) positioned 2 m in front of them at a height of 1.75 m. A rest period of 30 s was granted before the third exercise.

Exercise 3—Single-leg stance on a foam proprioceptive cushion: participants performed a single-leg stance on a flat-surface foam proprioceptive cushion. Three sets of 30 s were completed for each limb. The exercise performed with one limb corresponded to the rest period for the contralateral limb, without additional rest periods. During this exercise, a red dot (4 cm diameter) positioned 2 m in front of them at a height of 1.75 m, was visually fixated by the participants.

#### 2.3.2. Control Group (CG)

Participants allocated to the control group (CG) did not receive any form of training. To avoid any kind of stimulation of the plantar surface, they were positioned in a supine resting posture on a couch for the same amount of time as the experimental intervention.

### 2.4. Outcomes

The order of assessments was blocked as follows: ankle ROM (left and right) and the Y-Balance Test (YBT). For the ankle ROM, each participant’s right foot was assessed first, followed by the left foot. For the YBT, participants performed the test first with the right supporting limb and then with the left supporting limb.

Assessments were conducted before (T0) and immediately after (T1) the experimental or control condition. All measurements were performed by the same experimenter.

#### 2.4.1. Dynamic Balance

The dynamic balance was assessed using the YBT. The YBT was performed using the YBT kit ^TM^ (Move2Perform, Evansville, IN, USA), which consists of a central stance platform and three indicator boxes with movable reach indicators that record reach distance in centimeters [34].

Participants were instructed to maintain balance on one limb while reaching as far as possible with the contralateral limb in three directions (anterior, posteromedial, and posterolateral), maintaining their hands on their hips, maintaining their heel of the test/stance limb on the floor at all times, avoiding kicking, or slinging and returning the reach limb to the starting position without losing their balance.

Three trials were allowed for each reach direction on each limb. The maximum reach distance achieved in each direction was recorded. For each limb, a composite score was calculated using the following equation: composite score = [(sum of the greatest reach distances in the three directions)/(3 × limb length)] × 100 [35].

#### 2.4.2. Ankle ROM

Ankle ROM was assessed using an inertial sensor (Beyond Inertial, Motustech, Guidonia Montecelio, Rome, Italy) [36]. Ankle dorsiflexion and plantarflexion were assessed in a non-weight-bearing condition, with the participants positioned prone on a couch with both feet hanging out of the bed [37]. This position was selected to isolate ankle ROM. A band was placed around the participant’s calves, wrapping it around the couch to prevent compensatory movements with the lower limbs. To measure ankle ROM, the inertial sensor was placed on the participant’s metatarsus using an elastic band.

For each movement, three trials were recorded, and the mean value of the three trials was used for statistical analysis.

### 2.5. Randomization Process

The online randomization tool (randomizer.org) was used to generate the random allocation sequence. An experimenter generates the random allocation sequence. Participants were recruited voluntarily and enrolled before group assignment. Group allocation was performed after participant enrollment according to the predefined random sequence. No procedures were implemented to blind outcome assessment.

### 2.6. Statistical Analysis

Descriptive statistics (mean ± standard deviation) were calculated for participant characteristics, including age, height, and weight. Baseline comparisons between groups for age, height, and weight were performed using *t*-tests, with subgroup analyses by sex for each group.

Data distribution was assessed using the Shapiro–Wilk test. Statistical significance was set at *p* = 0.05.

For variables showing a normal distribution, repeated-measures analysis of variance (ANOVA) was conducted to evaluate within-group and between-group effects, and partial eta squared (η^2^*p*) was used to measure the effect size. Post hoc comparisons were performed using the Bonferroni correction. For variables not normally distributed, non-parametric Friedman test was applied. Kendall’s effect size (W) was calculated as χ^2^/N(k − 1) where χ^2^ is the Friedman test, N is the number of subjects, and k is the number of measurements. Pairwise comparisons were conducted using the Durbin–Conover test to assess within-group and between-group effects. All analyses were exploratory in nature and were conducted to preliminarily investigate outcome behavior and variability in this randomized pilot trial.

Statistical analyses were performed using Jamovi software (Version 2.6) [38].

## 3. Results

### 3.1. Participants

All participants received the allocated intervention and completed the study protocol. No dropouts or exclusions occurred after randomization. Therefore, all randomized participants were included in the final analysis.

**Figure 1:**
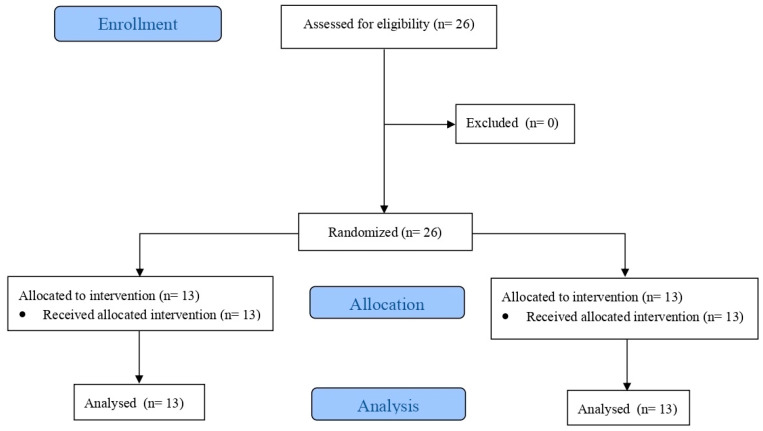
Participants flow diagram.

A total of 26 participants, allocated to either the EG (n = 13; five females and eight males) or the CG (n = 13; five females and eight males) were recruited. A flow diagram of participants’ progression through the study is presented in Figure 1.

Baseline demographic and anthropometric characteristics of participants are presented in Table 1. No statistical differences were observed between groups for all variables (*p* > 0.05), but subgroup analysis by sex showed within-group significance in the CG for weight (males = 73.1 ± 4.97 kg; females = 55.6 ± 9.15 kg; *p* < 0.001) and height (males = 175.9 ± 7.7 cm; females = 162 ± 6.2 cm; *p* = 0.006), and in the EG for age (males = 23.3 ± 0.463 years; females = 22 ± 0.707 years; *p* = 0.003), weight (males = 77.9 ± 18.404 kg; females = 55 ± 4.359 kg; *p* = 0.021), and height (males = 176.4 ± 7.19 cm; females = 160.2 ± 6.301 cm; *p* = 0.002).

### 3.2. Outcomes

#### 3.2.1. Dynamic Balance

For the primary outcome, that is, the dynamic balance, changes from T0 to T1 were analyzed within- and between-group. Concerning the YBT performed with the right lower limb (YBT RT), we conducted a parametric repeated measures ANOVA with Bonferroni post hoc correction, while for the YBT performed with the left lower limb (YBT LT), we conducted a non-parametric repeated measures ANOVA (Friedman) with pairwise comparisons (Durbin–Conover). Table 2 shows the YBT RT time*group and between-group Bonferroni post hoc test.

Table 3 shows the YBT RT time*group. We found significance within-group in the YBT RT. Specifically, significance was found for time (*p* = 0.032, F_(1,24)_ = 5.18, η^2^*p* = 0.178) and for time*group interaction (*p* = 0.001, F_(1,24)_ = 13.35, η^2^*p* = 0.357). Regarding the post hoc analysis, we found a significant difference only for the EG from T0 to T1 (*p* = 0.002). No between-group differences were found (*p* = 0.161, F_(1,24)_ = 2.09, η^2^*p* = 0.080).

Regarding YBT LT, the non-parametric repeated measures ANOVA (Friedman) revealed significance (*p* = 0.032, χ^2^ = 8.83, W = 0.34). Table 4 shows the pairwise comparisons (Durbin–Conover) for YBT LT, while Table 5 shows the medians from T0 to T1 for both conditions. For YBT LT, we found significance in the EG from T0 to T1 (*p* = 0.015).

#### 3.2.2. Ankle ROM

Concerning the ankle ROM, no significant changes were observed (*p* > 0.05).

No significant differences either within- or between-group were found for ankle dorsiflexion and plantarflexion.

## 4. Discussion

This pilot study aimed to examine the acute effects of plantar proprioceptive training on dynamic balance and ankle joint mobility in physically active young adults.

As reported in previous studies, ankle proprioception may be more developed in athletes as dancers, gymnasts, badminton players, soccer players, and swimmers, due to the years of sport-specific practice that may enhance the efficiency and reliability of proprioceptive processing at the central level [9,39,40,41,42].

The preliminary results of this study conducted in physically active young adults suggest that plantar proprioceptive training may positively influence dynamic balance performance, while no significant effects were observed on ankle ROM. The observed improvement in dynamic balance following plantar proprioceptive training aligns with previous findings reported by Yong et al. (2017), who reported enhanced dynamic balance after an ankle proprioceptive training program in healthy adults [43]. Indeed, proprioception provides essential sensory information to the CNS, contributing to joint position sense, segmental stability, and overall postural alignment [43]. Park et al. (2023) assessed dynamic balance in four different conditions: barefoot, minimalist shoes, regular athletic shoes with regular insoles, and regular athletic shoes with textured insoles [44]. They found improvements in the YBT with the regular athletic shoes with textured insoles, and this is in line with the scientific literature because both tactile and proprioception stimulation play an important role in balance [44].

The experimental protocol, which combined walking and balance exercises, was designed to replicate a plantar stimulation environment, in which plantar stimuli were integrated with motor and postural control. In this way, proprioceptive inputs from muscle and joint receptors, together with afferents from plantar dermis receptors, provide an integrated and multifaceted source of information, essential for maintaining balance [45,46,47,48,49,50,51]. Indeed, based on this sensory input, different types of orthopedic devices and sensory-stimulating shoes have been created with the aim of enhancing somatosensory feedback from the plantar surface to improve gait performance and postural stability [52,53,54]. In fact, previous studies showed the capacity of sensory-stimulating insoles and modified floor surfaces to influence body kinematics and kinetics during balance and gait-related tasks in healthy young adults, older adults, and individuals from clinical populations [52,53,55,56,57,58]. In this context, the observed improvements in dynamic balance may be explained by enhanced efficiency in sensory integration processes rather than structural or mechanical changes. Supporting this interpretation, no significant changes were observed in ankle ROM. Moreover, the absence of significant effects in ankle ROM may be due to the fact that the right and left ankle measurements were taken in the non-weight-bearing position.

This pilot study suggests that plantar proprioceptive training can provide acute improvements in dynamic balance performance, while no significant changes were observed in ankle ROM.

These findings are consistent with the hypothesis that plantar inputs can influence postural control mechanisms rather than inducing immediate changes in ankle mobility. Further studies with larger samples, longer intervention periods, and targeted assessments are needed to better isolate the specific contribution of plantar proprioceptive training to confirm its role on balance performance.

### 4.1. Limitations

This pilot study has several limitations. First of all, the small sample size does not allow for generalizations of the results. The open-label design may have introduced significant performance bias. It should be mentioned that the study was designed to evaluate the acute responses to plantar proprioceptive training rather than long-term adaptations, so the short duration of the intervention and the lack of follow-up assessments do not allow us to draw definitive conclusions. Moreover, since stimulating the plantar surface of the foot involves both the epidermis and the dermis, we are unable to determine whether the observed changes result from the stimulation of the dermis, epidermis, or both. Furthermore, the experimental protocol makes it difficult to isolate the specific contribution of proprioceptive stimulation from motor and postural control training effects because it combined walking and balance exercises. Another limitation concerns the broad inclusion criteria and the fact that baseline physical activity levels were not quantified. Although a complete neuro-otological visit was administered, oculomotor dysfunctions and temporomandibular joint disorders, which are also known to influence postural control, were not assessed. Another limit is represented by the CG, which remained lying supine on a couch to avoid any kind of stimulation of the plantar surface. This condition may have induced relaxation effects that could have influenced the results, thus representing a potential confounding factor. In addition, ankle ROM was assessed in a non-weight-bearing condition, and although this approach allows for better isolation of joint motion, it does not reflect the functional weight-bearing conditions to which ankle mobility is typically subjected.

### 4.2. Practical Implications

The findings of this study are primarily applicable to physically active young adults without postural, musculoskeletal, neurological, and vestibular disorders. We cannot draw definitive conclusions about the effects in clinical populations, sedentary individuals, or older adults. The feasibility of the protocol, the absence of negative effects, and the sensitivity of the measurements indicate that this plantar proprioceptive training could represent a simple and cost-effective strategy to enhance dynamic balance performance. In sports, it could be integrated into warm-up routines or injury prevention programs to improve postural control and potentially reduce the risk of lower limb injuries.

## Figures and Tables

**Table 1:** Characteristics of participants.

Variables	Group	Mean	SD
Age (years)	CG	23.2	1.625
	EG	22.8	0.832
Weight (kg)	CG	66.4	11.004
	EG	69.1	18.387
Height (cm)	CG	170.5	9.837
	EG	170.2	10.511

Legend. CG = Control group; EG = Experimental group; SD = Standard deviation.

**Table 2:** YBT RT time*group and between-group Bonferroni post hoc test.

Time*Group	*p*	Mean Difference	SE	t
T0 CG–T0 EG	1.000	−3.02	4.26	−0.708
T0 CG–T1 CG	1.000	1.21	1.24	0.974
T0 EG–T1 EG	0.002	−5.20	1.24	−4.193
T1 CG–T1 EG	0.287	−9.42	4.52	−2.086
**Between-group**	***p***	**Mean Difference**	**SE**	**t**
CG − EG	0.161	−6.22	4.30	−1.45

YBT RT = Y-Balance Test Right; CG = Control group; EG = Experimental group; SE = Standard error.

**Table 3:** YBT RT time*group.

Group	Time	Mean	SE
CG	T0	88.6	3.01
	T1	87.4	3.19
EG	T0	91.6	3.01
	T1	96.8	3.19

YBT RT = Y-Balance Test Right; CG = Control group; EG = Experimental group; SE = Standard error.

**Table 4:** YBT LT pairwise comparisons (Durbin–Conover).

Time*Group	*p*
T0 CG–T0 EG	0.693
T0 CG–T1 CG	0.160
T1 CG–T1 EG	0.138
T0 EG–T1 EG	0.015

YBT LT = Y-Balance Test Left; CG = Control group; EG = Experimental group.

**Table 5:** YBT LT medians from T0 to T1.

Group	Time	Median
CG	T0	88.6
	T1	91.0
EG	T0	91.3
	T1	98.3

YBT LT = Y-Balance Test Left; CG = Control group; EG = Experimental group.

## Data Availability

The raw data supporting the conclusions of this article will be made available by the authors on request.

## Abbreviations

The following abbreviations are used in this manuscript:

TPS	Tonic Postural System
CNS	Central Nervous System
CAI	Chronic Ankle Instability
ROM	Range of Motion
EG	Experimental group
CG	Control group
YBT	Y-Balance Test
ANOVA	Analysis of Variance
YBT RT	Right Lower Limb
YBT LT	Left Lower Limb

## References

[1] Oravitan M. (2009). Posturology-fundamental concepts and practical applications. Analele UVT-Ser. EFS.

[2] Carini F., Mazzola M., Fici C., Palmeri S., Messina M., Damiani P., Tomasello G. (2017). Posture and posturology, anatomical and physiological profiles: Overview and current state of art. Acta Biomed..

[3] Scoppa F. (2002). Posturology: The neurophysiological model, the biomechanical model, the model psychosomatic. Otoneurologia.

[4] Rowe M., Iwamura Y. (2001). Somatosensory Processing: From Single Neuron to Brain Imaging.

[5] Kam P., Power I. (2020). Principles of Physiology for the Anaesthetist.

[6] Macintyre P., Rowbotham D., Walker S. (2008). Clinical Pain Management: Acute Pain.

[7] Hwang S., Agada P., Kiemel T., Jeka J.J. (2016). Identification of the Unstable Human Postural Control System. Front. Syst. Neurosci..

[8] Diener H.C., Dichgans J. (1988). On the role of vestibular, visual and somatosensory information for dynamic postural control in humans. Prog. Brain Res..

[9] Han J., Waddington G., Adams R., Anson J., Liu Y. (2016). Assessing proprioception: A critical review of methods. J. Sport Health Sci..

[10] Goble D.J. (2010). Proprioceptive acuity assessment via joint position matching: From basic science to general practice. Phys. Ther..

[11] Pasma J.H., Boonstra T.A., Campfens S.F., Schouten A.C., Van der Kooij H. (2012). Sensory reweighting of proprioceptive information of the left and right leg during human balance control. J. Neurophysiol..

[12] Bouisset S., Do M.C. (2008). Posture, dynamic stability, and voluntary movement. Neurophysiol. Clin..

[13] Clark N.C., Röijezon U., Treleaven J. (2015). Proprioception in musculoskeletal rehabilitation. Part 2: Clinical assessment and intervention. Man. Ther..

[14] Felten D.L., O’Banion M.K., Maida M.S., Felten D.L., O’Banion M.K., Maida M.S. (2016). 9—Peripheral Nervous System. Netter’s Atlas of Neuroscience.

[15] Selva-Sarzo F., Sanchez Romero E.A., Cuenca-Zaldivar J.N., Garcia-Haba B., Akiyama C., Sillevis R., Fernandez-Carnero S. (2025). Effects on perceived pain and somatosensory function after transcutaneous neuromodulation in patients with chronic low back pain: A quasi-experimental study with a crossover intervention. Front. Pain Res..

[16] Brown T.M., Krishnamurthy K. (2026). Histology, Dermis. StatPearls.

[17] Lopez-Ojeda W., Pandey A., Alhajj M., Oakley A.M. (2026). Anatomy, Skin (Integument). StatPearls.

[18] Yousef H., Alhajj M., Fakoya A.O., Sharma S. (2025). Anatomy, Skin (Integument), Epidermis.

[19] Corbin D.M., Hart J.M., McKeon P.O., Ingersoll C.D., Hertel J. (2007). The effect of textured insoles on postural control in double and single limb stance. J. Sport Rehabil..

[20] Kavounoudias A., Roll R., Roll J.P. (1998). The plantar sole is a ‘dynamometric map’ for human balance control. Neuroreport.

[21] Wang Y., Watanabe K., Chen L. (2016). Effect of plantar cutaneous inputs on center of pressure during quiet stance in older adults. J. Exerc. Sci. Fit..

[22] Wang Y., Zatsiorsky V.M., Latash M.L. (2006). Muscle synergies involved in preparation to a step made under the self-paced and reaction time instructions. Clin. Neurophysiol..

[23] Hlavackova P., Vuillerme N. (2012). Do somatosensory conditions from the foot and ankle affect postural responses to plantar-flexor muscles fatigue during bipedal quiet stance?. Gait Posture.

[24] Ruhe A., Fejer R., Walker B. (2011). Center of pressure excursion as a measure of balance performance in patients with non-specific low back pain compared to healthy controls: A systematic review of the literature. Eur. Spine J..

[25] Abraira V.E., Ginty D.D. (2013). The sensory neurons of touch. Neuron.

[26] Inglis J.T., Kennedy P.M., Wells C., Chua R. (2002). The role of cutaneous receptors in the foot. Adv. Exp. Med. Biol..

[27] Kennedy P.M., Inglis J.T. (2002). Distribution and behaviour of glabrous cutaneous receptors in the human foot sole. J. Physiol..

[28] Kavounoudias A., Roll R., Roll J.P. (1999). Specific whole-body shifts induced by frequency-modulated vibrations of human plantar soles. Neurosci. Lett..

[29] Priplata A.A., Niemi J.B., Harry J.D., Lipsitz L.A., Collins J.J. (2003). Vibrating insoles and balance control in elderly people. Lancet.

[30] Zehr E.P., Collins D.F., Chua R. (2001). Human interlimb reflexes evoked by electrical stimulation of cutaneous nerves innervating the hand and foot. Exp. Brain Res..

[31] Zehr E.P., Stein R.B., Komiyama T. (1998). Function of sural nerve reflexes during human walking. J. Physiol..

[32] Fakontis C., Iakovidis P., Kasimis K., Lytras D., Koutras G., Fetlis A., Algiounidis I. (2023). Efficacy of resistance training with elastic bands compared to proprioceptive training on balance and self-report measures in patients with chronic ankle instability: A systematic review and meta-analysis. Phys. Ther. Sport.

[33] Han J., Luan L., Adams R., Witchalls J., Newman P., Tirosh O., Waddington G. (2022). Can Therapeutic Exercises Improve Proprioception in Chronic Ankle Instability? A Systematic Review and Network Meta-analysis. Arch. Phys. Med. Rehabil..

[34] Nozu S., Johnson K.A., Tanaka T., Inoue M., Nishio H., Takazawa Y. (2023). The Accuracy of Ankle Eccentric Torque Control Explains Dynamic Postural Control During the Y-Balance Test. Int. J. Sports Phys. Ther..

[35] Cook G., Plisky P. (2010). Y Balance Test Manual. Functional Movement Systems.

[36] Belmonte G., Canzone A., Gervasi M., Fernández-Peña E., Iovane A., Bianco A., Patti A. (2025). Sufficient Standardization? Evaluating the Reliability of an Inertial Sensor (Beyond^TM^) for Ankle Dorsiflexion After a Brief Familiarization Period. Sports.

[37] Thomas E., Scardina A., Pinto G., Serafini S., Nakamura M., Konrad A., Campa F., Bellafiore M., Bianco A. (2025). Determinants of acute effects of stretching vs. foam rolling: Morphological, sensory and fluid responses. J. Bodyw. Mov. Ther..

[38] (2024). The Jamovi Project. Jamovi.

[39] Han J., Anson J., Waddington G., Adams R. (2014). Sport attainment and proprioception. Int. J. Sports Sci. Coach..

[40] Aligene K., Lin E. (2013). Vestibular and balance treatment of the concussed athlete. NeuroRehabilitation.

[41] Aydin T., Yildiz Y., Yildiz C., Atesalp S., Kalyon T.A. (2002). Proprioception of the ankle: A comparison between female teenaged gymnasts and controls. Foot Ankle Int..

[42] Aydin T., Yildiz Y., Yildiz C., Kalyon T.A. (2002). Effects of extensive training on female teenage gymnasts’ active and passive ankle-joint position sense. J. Sport Rehabil..

[43] Yong M.S., Lee Y.S. (2017). Effect of ankle proprioceptive exercise on static and dynamic balance in normal adults. J. Phys. Ther. Sci..

[44] Park J.H., Benson R.F., Morgan K.D., Matharu R., Block H.J. (2023). Balance effects of tactile stimulation at the foot. Hum. Mov. Sci..

[45] Macefield G., Burke D., Gandevia S.C. (1989). The cortical distribution of muscle and cutaneous afferent projections from the human foot. Electroencephalogr. Clin. Neurophysiol..

[46] Macefield V.G. (2021). The roles of mechanoreceptors in muscle and skin in human proprioception. Curr. Opin. Physiol..

[47] Knellwolf T.P., Burton A.R., Hammam E., Macefield V.G. (2018). Microneurography from the posterior tibial nerve: A novel method of recording activity from the foot in freely standing humans. J. Neurophysiol..

[48] Strzalkowski N.D.J., Peters R.M., Inglis J.T., Bent L.R. (2018). Cutaneous afferent innervation of the human foot sole: What can we learn from single-unit recordings?. J. Neurophysiol..

[49] Viseux F.J.F. (2020). The sensory role of the sole of the foot: Review and update on clinical perspectives. Neurophysiol. Clin..

[50] Van Wezel B.M., Ottenhoff F.A., Duysens J. (1997). Dynamic control of location-specific information in tactile cutaneous reflexes from the foot during human walking. J. Neurosci..

[51] Nurse M.A., Nigg B.M. (2001). The effect of changes in foot sensation on plantar pressure and muscle activity. Clin. Biomech..

[52] Hijmans J.M., Geertzen J.H., Zijlstra W., Hof A.L., Postema K. (2008). Effects of vibrating insoles on standing balance in diabetic neuropathy. J. Rehabil. Res. Dev..

[53] Priplata A.A., Patritti B.L., Niemi J.B., Hughes R., Gravelle D.C., Lipsitz L.A., Veves A., Stein J., Bonato P., Collins J.J. (2006). Noise-enhanced balance control in patients with diabetes and patients with stroke. Ann. Neurol..

[54] Perry S.D., Radtke A., McIlroy W.E., Fernie G.R., Maki B.E. (2008). Efficacy and effectiveness of a balance-enhancing insole. J. Gerontol. A Biol. Sci. Med. Sci..

[55] Moon J., Pathak P., Kim S., Roh S.G., Roh C., Shim Y., Ahn J. (2020). Shoes with active insoles mitigate declines in balance after fatigue. Sci. Rep..

[56] Kenny R.P.W., Eaves D.L., Martin D., Hatton A.L., Dixon J. (2019). The effects of textured insoles on quiet standing balance in four stance types with and without vision. BMC Sports Sci. Med. Rehabil..

[57] Hatton A.L., Williams K., Chatfield M.D., Hurn S.E., Maharaj J.N., Gane E.M., Cattagni T., Dixon J., Rome K., Kerr G. (2023). Immediate effects of wearing textured versus smooth insoles on standing balance and spatiotemporal gait patterns when walking over even and uneven surfaces in people with multiple sclerosis. Disabil. Rehabil..

[58] Ma C.C., Rao N., Muthukrishnan S., Aruin A.S. (2018). A textured insole improves gait symmetry in individuals with stroke. Disabil. Rehabil..

